# Supplementary material for: Spatial variation and attributable risk factors of anaemia among young children in Uganda: Evidence from a nationally representative survey
Source: PLOS Glob Public Health. 2023 May 17;3(5):e0001899. doi: 10.1371/journal.pgph.0001899 (PMC10191331; doi:10.1371/journal.pgph.0001899)
Supplement: S1 Table — (DOCX) [file pgph.0001899.s001.docx]

**S1 Table**: Hot spot and cold spot analysis of anaemia among enumeration areas (clusters) per sub-region

| Sub-region | Total number of communities | Hot spots | Cold spots | Non-significant communities |
| --- | --- | --- | --- | --- |
| Acholi | 39 | 23 | 0 | 16 |
| Ankole | 48 | 0 | 39 | 9 |
| Bugisu | 39 | 0 | 33 | 6 |
| Bukedi | 41 | 0 | 13 | 28 |
| Bunyoro | 44 | 2 | 7 | 35 |
| Busoga | 59 | 21 | 2 | 36 |
| North Buganda | 67 | 5 | 2 | 60 |
| South Buganda | 58 | 5 | 7 | 46 |
| Kampala | 44 | 0 | 0 | 44 |
| Karamoja | 33 | 12 | 1 | 20 |
| Kigezi | 39 | 0 | 38 | 1 |
| Lango | 44 | 16 | 0 | 28 |
| Teso | 40 | 7 | 4 | 29 |
| Tooro | 48 | 0 | 21 | 27 |
| West Nile | 45 | 17 | 2 | 26 |
