# Supplementary material for: Spatial variation and attributable risk factors of anaemia among young children in Uganda: Evidence from a nationally representative survey
Source: PLOS Glob Public Health. 2023 May 17;3(5):e0001899. doi: 10.1371/journal.pgph.0001899 (PMC10191331; doi:10.1371/journal.pgph.0001899)
Supplement: S2 Table — (DOCX) [file pgph.0001899.s002.docx]

**S2 Table:** SaTScan analysis result of hot spot areas of anaemia among children in Uganda

| **Clusters** | **Districts identified** | **Population** | **Cases** | **RR** | **LLR** | **p-value** |
| --- | --- | --- | --- | --- | --- | --- |
| 1(226)  Primary | West Nile (Koboko, Maracha, Moyo, Adjumani, Yumbe, Nebbi)  Acholi (Agago, Omoro, Amuru, Gulu, Pader, Lamwo, Amuru)  Karamoja (Kaabong, Kotido, Abim, Moroto, Napiripirit)  Lango (Oyam, Amolatar, Otuke, Apac, Dokolo, Lira, Alebtong)  Teso (Amuria, Kumi, Soroti, Katakwi, Ngora)  Bunyoro (Kiryandongo, Masindi) | 1446 | 921 | 1.32 | 43.058 | 0.000 |
| 2(4)  Secondary | Kayunga and Kamuli | 22 | 22 | 1.86 | 13.559 | 0.001 |
| Other clusters |  |  |  |  |  |  |
| 3(13) | Buikwe, Buvuma | 81 | 59 | 1.36 | 6.113 | >0.05 |
| 4(1) | Kagadi | 9 | 9 | 1.85 | 5.534 | >0.05 |
| 5(2) | Bundibudgyo | 14 | 13 | 1.72 | 5.173 | >0.05 |
| 6(11) | Mayuge, Namayingo | 53 | 39 | 1.37 | 4.308 | >0.05 |
| 7(1) | Bukomansimbi | 7 | 7 | 1.85 | 4.303 | >0.05 |

RR = Relative Risk, LLR = Log-Likelihood Ratio.
