# Supplementary material for: Spatial variation and attributable risk factors of anaemia among young children in Uganda: Evidence from a nationally representative survey
Source: PLOS Glob Public Health. 2023 May 17;3(5):e0001899. doi: 10.1371/journal.pgph.0001899 (PMC10191331; doi:10.1371/journal.pgph.0001899)
Supplement: Attachment — Submitted filename: Response_to_reviewers.pdf [file pgph.0001899.s003.pdf]

POINT BY POINT RESPONSE TO THE REVIEWERS

Reviewer #1

The authors conducted a cross-sectional study utilizing the 2016 Uganda Demographic and Health Survey (UDHS) data to evaluate the spatial variation and preventable risk factors of anaemia among children aged 6-59 moths in Uganda. The findings of the study provide comprehensive evidence on the spatial variation of anaemia prevalence and the associated risk factors among children aged 6-59 months in Uganda. The authors provide a well-written paper with thoughtful analysis and discussion. Regarding the research strategy and findings, reviewers have several comments that should be addressed prior to publication.

Abstract

The results and conclusions revolve around the spatial variation and risk factors of anaemia in Uganda, however, the background rationale doesn't touch on these a priori relationships. It would be helpful to revise the abstract background with a concept focusing on the research gap (the 3rd-5th paragraph of the Introduction).

Action:

Page 1: Abstract-We appreciate the reviewer for this observation and guidance. We have modified the introduction in the abstract to focus on the research gap.

Introduction

1. The prevalence and factors associated with anaemia in sub-Saharan African countries have been largely investigated, the rationale for study purpose would been benefited from the extended discussion of these observations (ref 8-12).

Action: We thank the reviewer for this suggestion. We have further discussed the factors associated with anaemia (Introduction, Page3-4)

Methods

A few additional pieces of information included in the methods would help contextualize the results.

1) Is the sample of 20,880 households national representative? Please further explain.

Action: We thank the review for the question. The sample size of 20,880 households is indeed a national representative. During the 2016 UDHS, a total of 696 which are distributed across the country were selected using the 2014 Uganda National Population and Housing Census. Each EA had a list of households that were selected systematically. In total, 30 households were selected in

each EA to give a total of 20,880 households for the 2016 UDHS. This has been explained further in the manuscript.

2) The citations from UDHS or previous UDHS papers would be helpful to introduce the data source and sampling strategy.

Action: We appreciate the reviewer for this observation. Citation of 2016 UDHS has been made within the sampling strategy.

3) It would be helpful to list all investigated exposure at the beginning of the paragraph (Exposure variables).

Action: We thank the reviewer for this suggestion. This has been modified as to the guidance. All the exposure variables have been listed.

4) The extended description for how sample was weighted would be beneficial for data analysis.

Action: We thank the reviewer for this advice. We generated a weighting variable using the sample weight variable in the DHS divided by 1,000,000. We have added this text in the analysis.

Results

Both the number and the percentage are expected to be descripted in the text.

Action: We thank the reviewer for this suggestion. However, following the journal guidelines we thought important to only present percentages in the text.

Discussion

It is worthwhile to further discuss why primary clusters mainly in West Nile and other regions, and proposing the possible public health recommendations regarding the observation to decrease the prevalence of anaemia in these areas is expected (2nd paragraph and 9th paragraph in Discussion).

Action: We thank the reviewer for this insightful observation and suggestion. This has been modified as follows: The differences in the multidimensional poverty levels across regions partly explains this variation. First, according to the current national estimates, Northern region is the poorest part of the country with Karamoja leading followed by Acholi and West Nile sub-regions [31, 32]. As of 2020, an estimated 85%, 64%, 59%, and 57% of children in Karamoja, Acholi, West Nile, and Lango live in households below the national poverty line [32]. This region is also arid/semi-arid and therefore food crop growing is a challenge. Next to the north in deprivation, is

the eastern part of the country. The study showed that Busoga region has the highest risk of anaemia in the east. Not only being poor, land in this region is mainly used for cultivation of sugarcane and food crops growing is on a very small scale [33]. In addition, income generated from cultivation is inadequate for the population to be able to purchase food to meet all its needs. This scenario is totally different from Ankole or Kigezi regions which are food-baskets of the country. This therefore explains the regional differences of anaemia in this study. Thus, policies targeting poverty alleviation, climate change adaptation and food security (including integration of cash crop growing into food production) for sustainable livelihood should be implemented.

Tables and Figures

1. The unit (months or years) of age is expected to add in Table 1.

Action: Months have been included as guided.

2. There are a significant number of Figures. It is suggested to put some of them as supplemental materials.

Action: We thank the reviewer of this guidance. The paper has been revised and the number of figures reduced.

Reviewer #2

"Spatial variation and preventable risk factors of anaemia among young children in Uganda: evidence from a nationally representative survey" is an excellent piece of research manuscript in field of child health of Africa. I don't have any further queries regarding the objective, methods and analysis of this research as these are chosen very carefully. There is no problem in language and coherence as well from my perspective. Therefore, I suggest to accept this manuscript for the publication. However, the authors should answer the following question before the acceptance of manuscript.

What is the reason to choose the Cluster and outlier analysis and Hotspot analysis and even SaTScan Analysis?

Action: We appreciate the reviewer for this inquiry. Spatial analysis is sequential. It is very important to have all the analyses performed. The paper has been revised and some analyses that looks to be similar combined and not presented in the results. None the less Cluster and outlier analyses aimed at checking the nature of the entire study area. This approach helps to identify areas high anaemia prevalence and those with low. Hotspot analysis further identifies specific individual cluster locations that exhibit higher prevalence of anaemia. SaTScan Analysis on the other hand was intended to identify the most affected districts after scanning the entire study area. Unlike the previous approaches, this performs a log-likelihood test and relative risk ratios as well as p-values for the most affected areas generated.

If all these analyses are necessary discuss on the results obtained from these methods?

Action: We thank the reviewer for this suggestion. A discussion on all these has been provided.

I also expect to have some discussion on Spatial interpolation.

Action: We thank the reviewer for this suggestion. A discussion on spatial interpolation has been provided.

I also suggest similar analysis from other regions of the world. For example:

https://www.mdpi.com/1660-4601/19/14/8664

Action: We thank the reviewer for this suggestion. All the methods adopted in this study have also been used elsewhere including the proposed article (https://www.mdpi.com/1660-4601/19/14/8664).

Reviewer #3

Thank you for the opportunity to review this manuscript. The authors aimed to investigate the spatial variation and preventable risk factors of anaemia among children aged 6-59 months in Uganda. The manuscript is very well written and easy to follow. The objectives were clearly met using appropriate and thorough statistical methods. The authors are commended for their efforts. I recommend minor revision based on a few very minor grammatical errors in the manuscript.

Action: We appreciate the reviewer for the time given to this paper. Paper has been proofread and all identified grammatical errors corrected.

In addition, the authors should specify more clearly the random effect incorporated into the GLMM. Based on the discussion of the results, it appears to be based on the cluster. However, when describing the multilevel mixed effect model, this is not made clear.

Action: We thank the reviewer for this observation. This has been made clear. We ran a multilevel logistic regression with children at level 1 being nested within clusters (communities) at level 2. Communities were also nested within sub-regions. Therefore, the random part for each of the models both at regional and communities within regions was presented.
